# LCZ696 (sacubitril/valsartan), an angiotensin receptor neprilysin inhibitor (ARNI): clinical development in heart failure

**DOI:** 10.1186/2050-6511-16-S1-A1

**Published:** 2015-09-02

**Authors:** Martin P Lefkowitz

**Affiliations:** 1 Cardiovascular Metabolism, Novartis Pharmaceuticals Corporation, East Hanover, NJ 10901, USA

## 

Neurohormonal pathways are of critical importance in the pathogenesis and progression of heart failure. Current heart failure therapies mainly focus on blocking the detrimental effects of long-term neurohormonal activation and largely ignore the physiological compensatory effect of the natriuretic peptide system and other endogenous vasodilator systems. Natriuretic peptides exert their effects by activating membrane-bound guanylyl cyclase-coupled receptors (NPR-A and -B), resulting in increased concentrations of the second messenger cyclic guanosine monophosphate (cGMP) [1].

LCZ696 (sacubitril/valsartan) exhibits the novel mechanism of action of an angiotensin receptor neprilysin inhibitor (ARNI) by simultaneously inhibiting neprilysin (neutral endopeptidase; NEP) via LBQ657, the active metabolite of the prodrug sacubitril (AHU377), and blocking the angiotensin II type-1 receptor via valsartan [2]. The cardiovascular effects of LCZ696 in heart failure patients are attributed to the enhancement of peptides that are degraded by neprilysin such as natriuretic peptides by LBQ657 and the simultaneous inhibition of the deleterious effects of angiotensin II by valsartan.

In the PARADIGM-HF study of 8442 patients with heart failure and reduced ejection fraction (HFrEF), LCZ696 reduced the primary endpoint of cardiovascular death or heart failure hospitalization by 20% and reduced death due to any cause by 16% (both p<0.001) compared with the ACE-inhibitor enalapril. Symptoms and physical functioning, as assessed by the Kansas City Cardiomyopathy Questionnaire (patient assessment) and NYHA classification (physician assessment), were also favourably impacted by LCZ696 vs. enalapril. LCZ696 increased levels of cGMP and BNP while decreasing NT-proBNP and troponin levels (all p<0.001) [3,4].

LCZ696 is currently under investigation in patients with heart failure with preserved ejection fraction (HFpEF). Of note, hypertrophy signalling, diastolic relaxation and stiffness, and vasorelaxation are favourably modified by cGMP-dependent protein kinase phosphorylation, suggesting that agents that raise cGMP levels may have a role in the treatment of HFpEF [5]. In the proof-of-concept Phase II PARAMOUNT study (n=301), LCZ696 reduced NT-proBNP (primary endpoint) to a greater extent than did valsartan at 12 weeks (p<0.01), and was associated with left atrial reverse remodelling and improvement in NYHA class at 36 weeks (p<0.05) [6]. The PARAGON study is a large ongoing Phase III outcomes study (n=4300) that will clarify the role of LCZ696 in the treatment HFpEF, a condition for which there is currently no evidence-based therapy [7].
